# CDCA8 expression and its clinical relevance in patients with bladder cancer

**DOI:** 10.1097/MD.0000000000011899

**Published:** 2018-08-24

**Authors:** Yaqiong Bi, Song Chen, Jiazhi Jiang, Jie Yao, Gang Wang, Qiang Zhou, Sheng Li

**Affiliations:** a Department of Biological Repositories; b Department of Urology; c Laboratory of Precision Medicine, Zhongnan Hospital of Wuhan University, Wuhan, China.

**Keywords:** bladder cancer, CDCA8, prognosis

## Abstract

Cell division cycle associated 8 (CDCA8) overexpression is detected in various malignant tumors and closely associated with tumor growth. However, the correlations of CDCA8 expression with clinicopathological factors and prognosis of bladder cancer (BC) remain unclear. The purpose of this study was to identify the expression of CDCA8 and its clinical relevance in BC patients.

GEO datasets were employed to obtain CDCA8 expression data and its clinical information in BC samples. Real-time PCR (RT-PCR) was performed to detect the expression of CDCA8 in BC and the adjacent normal tissues. Nonpaired *t* test was used to statistically analyze the difference between the 2 groups. Cox univariable and multivariable analyses of overall survival (OS) and cancer specific survival (CSS) among BC patients were performed. Biological processes or signaling pathways that might mediate the activity of CDCA8 in BC were analyzed.

CDCA8 levels were significantly higher in BC (8.870 ± 0.08281 vs 7.472 ± 0.07035, *P < *.0001). CDCA8 expression was significantly associated with tumor progression (*P = *.001), T stage (*P < *.0001), N stage (*P = *.013), and grade (*P < *.0001). Higher expression of CDCA8 predicted poor cancer-specific survival (*P* < .0001, HR = 0.2752, 95% CI:0.1364-0.5554) and overall survival (*P* < .0001, HR = 0.4270, 95% CI: 0.2630–0.6930) in patients with BC. Cox univariable and multivariable analyses showed that intravesical therapy, N stage and progression were the independent influence factors of overall survival among bladder cancer patients, CDCA8 expression, tumor grade and progression were the independent influence factors of cancer specific survival among bladder cancer patients. The results of GSEA indicated that CDCA8-regulated gene sets associated with spermatogenesis, G2M checkpoint, E2F targets, Myc targets, mTORC1 signaling, mitotic spindle angiogenesis, PI3K/AKT/mTOR signaling, cholesterol homeostasis and glycolysis. Finally, RT-PCR results confirmed that CDCA8 expression was upregulated in BC (*P = *.0039).

CDCA8 is overexpressed in BC and its high levels are correlated with poor clinicopathological features of BC patients. Therefore, CDCA8 may act as a novel prognostic marker and therapeutical target in the diagnosis and treatment of patients with BC.

## 1 Introduction

Bladder cancer (BC) is one of the most common malignancies globally,^[1]^ and its prognosis remains poor due to the high rates of recurrence and metastasis.^[2,3]^ Generally, an imbalance between cell growth and apoptosis makes a major contribution to tumor growth.^[4]^ Cell division is necessary for normal tissue growth and development, but inappropriate cell division and chromosomal segregation may cause cell over-proliferation and finally lead to cancer.^[5]^ Cancer cells are usually aneuploid which results from cell division errors.^[6]^ Prior research suggested that cell cycle disorder correlated to cancer occurrence.^[7]^

Cell division cycle associated 8 (CDCA8), also known as Borealin/Dasra B, is a member of the chromosomal passenger complex (CPC) indispensable for transmission of the genome during cell division.^[8]^ During cytokinesis, the CPC localizes to the inner centromeres, promotes midzone organization, regulates furrow contractility, and specifies the cleavage plane.^[9–11]^ Therefore, the CDCA proteins play an important role in mitosis, intersecting chromosome segregation and cell division with cancer.^[12]^ In fact, CDCA8 is transcriptionally activated in human embryonic stem cells (hESCs) and cancer cells, but slightly, or even absently, expressed in normal tissues. Previous studies demonstrated that overexpression of CDCA8 was required for cancer growth and progression.^[13]^

In the present study, the study primary aim was that analyzing CDCA8 expression and its clinical relevance in BC patients. Secondly, biological processes or signaling pathways that might mediate the activity of CDCA8 in BC were analyzed as well.

## 2 Material and methods

### 2.1 Dataset collection of BC

Gene expression data of BC was downloaded from Gene Expression Omnibus (GEO) (http://www.ncbi.nlm.nih.gov/geo). Clinical data related to the GSE13507 dataset (N = 165)^[14,15]^ were obtained, in which the corresponding probe for the CDCA8 gene was ILMN_1709294.

### 2.2 Clinicopathological features research methods

All samples were categorized into 2 groups according to CDCA8 expression levels in the dataset (GSE13507), namely high expression group (>median value) and low expression group (<median value). Then we analyzed the relationship between the clinicopathological characteristics of BC patients and the prognostic value of CDCA8 in BC.

### 2.3 Gene set enrichment analysis (GSEA)

GSEA,^[16,17]^ a computationally biological method, is routinely disseminated to determine whether a priori defined set of genes shows statistically significant and concordant differences between 2 groups.^[18]^ BC samples in GSE13507 were classified into CDCA8 high expression group and CDCA8 low expression group as mentioned above. The association between CDCA8 and biological processes or signaling pathway gene sets was analysed using GSEA v3.0 (http://www.broad.mit.edu/gsea/) with reference to gene sets from the Molecular Signatures Database (MSigDB). Thresholds for significance were determined by permutation analysis (1,000 permutations). Enrichment results with a false discovery rate (FDR) < 0.25 and a nominal *P*-value < .05 were considered statistically significant.

### 2.4 Patients and samples

Human BC tissue samples (n = 10) and the adjacent normal bladder tissues (n = 10) were collected from patients suffering BC surgery at Zhongnan Hospital of Wuhan University between October 10, 2016 and October 10, 2017. The fresh samples were stored in liquid nitrogen before use. In this work, the tissue specimens and clinical materials used in this study were collected after each participant gave written informed consent based on our institutional ethical guidelines. The Medical Ethics Committee, Zhongnan Hospital of Wuhan University approved the utilization of tumor tissues for this study.

### 2.5 RNA isolation and reverse transcription

Total RNA was isolated from collected bladder tissues using Qiagen RNeasy Mini Kit (Cat.#74101, Qiagen Ltd., Germany) and QIAshredder (Cat. #79654, Qiagen Ltd., Germany) according to the manufacturer's protocol. DNase I (RNase-Free DNaseSet, Cat. #79254, Qiagen Ltd., Germany) was used to remove contamination of gDNA from the RNA samples. Consent ration of isolated RNA was measured by Nano Photometer (Cat. #N60, Implen Ltd., Germany). Total RNA (1 μg) isolated from bladder tissues was mixed with oligo (dT) 12 to 18 primers to synthesize first-strand cDNA by using Revert Aid First Strand cDNA Synthesis Kit (Thermo Scientific, China).

### 2.6 Quantitative real time PCR (qRT-PCR) Analysis

cDNA (1 μg) was used for each reaction of the polymerase chain reactions (PCR) with a final volume of 20 μL. All primers conducted with the SYBR Premix Ex Taq II (Takara Bio, China) were tested for optimal annealing temperatures and PCR conditions were optimized with gradient PCRs on a Bio-Rad iCycler (Cat. #CFX96). Primer sequences and annealing temperatures were summarized in Table 1. Glyceraldehyde-3-phosphate dehydrogenase (GAPDH) alleles were used as internal reference. Relative gene abundance = 2−^ΔΔct^, Δct = ct_targe gene_ − ct_GAPDH_, for bladder tissues ΔΔct = Δct_BCa tissues_–Δct_normal bladder tissues_ (ct = threshold cycle).

**Table 1:**
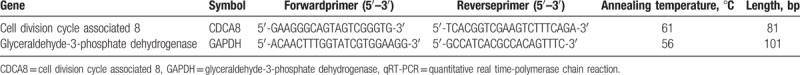
List of primers for qRT-PCR.

## 3 Statistical analysis

All statistical analyses were performed using SPSS statistical software (version 21.0) and GraphPad Prism (version 6.0). Each experiment was repeated 3 times, and all results were presented as the means ± standard deviation (
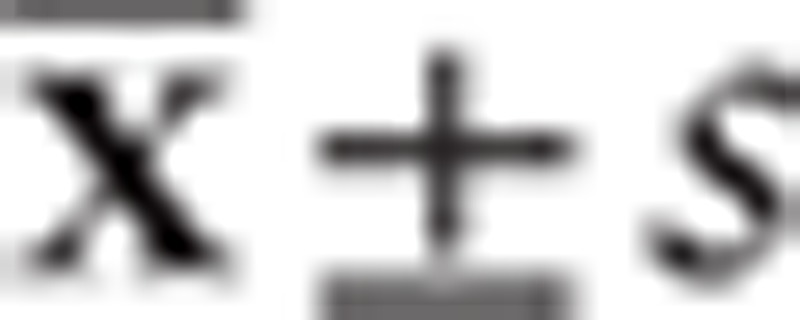
). Nonpaired *t* test was employed to analyze the difference in 2 groups regarding the expression of CDCA8 in BC, and Chi-square test was used to evaluate the correlations between CDCA8 expression and the clinicopathologic features of BC patients. Additionally, log-rank test was used to analyze the overall survival and cancer-specific survival in patients with BC. Kaplan–Meier plots were generated for the survival analysis. Cox univariable and multivariable analyses of overall survival (OS) and cancer specific survival (CSS) among BC patients were performed. *P < *.05 was considered statistically significant. Additionally, the associated gene sets regulated by CDCA8 were explored using GSEA.

## 4 Results

### 4.1 The expression of CDCA8 in BC

**Figure 1:**
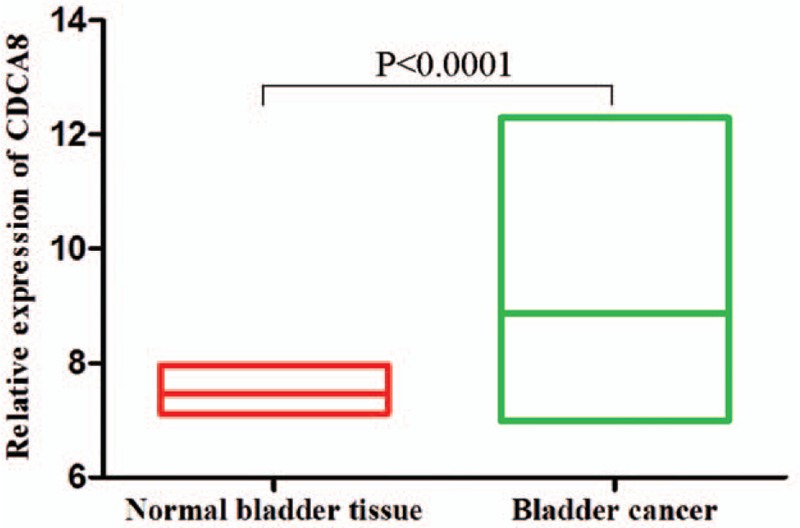
The expression levels of CDCA8 in bladder cancer and normal bladder tissues. These data were downloaded from Gene Expression Omnibus (GEO) of National Center for Biotechnology Information (NCBI) Clinical data related to the GSE13507 dataset (N = 165). CDCA8 = cell division cycle associated 8, GEO =  Gene Expression Omnibus, NCBI = National Center for Biotechnology Information.

Expression values of CDCA8 in normal tissues and BC cells were evaluated using BC gene expression profile study GSE13507 (*N* = 165). As shown in Figure 1, the expression of CDCA8 was significantly elevated in BC tissues compared with that in the normal tissues (7.472 ± 0.07035 vs 8.870 ± 0.08281, *P < *.0001).

### 4.2 The correlation between CDCA8 expression and clinical characteristics of BC patients

To investigate the correlation between CDCA8 expression and clinical characteristics of BC patients, the clinical data of 165 patients was collected, referring to progression, T stage, N stage, M stage, and grade. The results showed that CDCA8 expression in tumor tissues was significantly associated with progression (*P = *.001), T stage (*P < *.0001), N stage (*P = *.013), and grade (*P < *.0001) (Table 2). These results suggested that high expression of CDCA8 might be associated with tumor aggression and progression of BC.

**Table 2:**
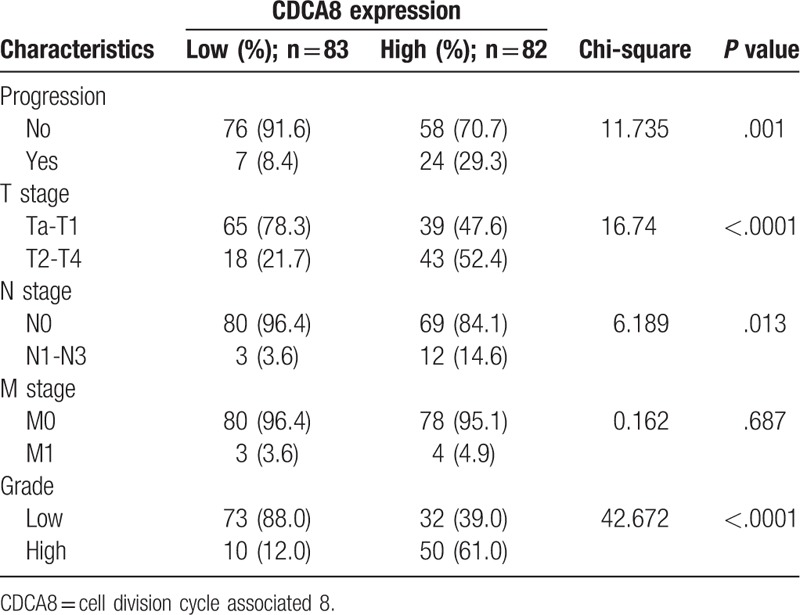
Associations between CDCA8 expression and clinicopathological factors of patients with bladder cancer.

### 4.3 The correlation between CDCA8 expression and prognosis of BC patients

**Figure 2:**
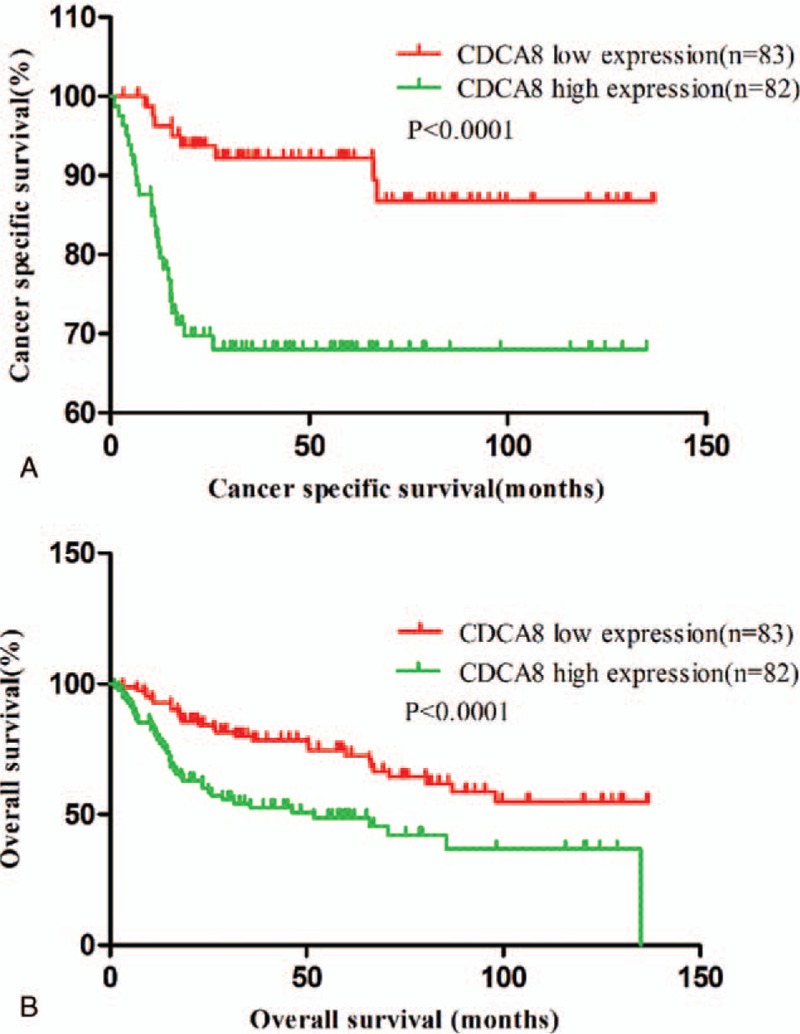
Associations between CDCA8 expression and patient's cancer-specific survival (A) and overall survival (B). CDCA8 = cell division cycle associated 8.

Significant association was observed between high expression of CDCA8 and shorter cancer-specific survival (*P < *.0001, HR = 0.2752, 95% CI: 0.1364-0.5554, Fig. 2A), so was the case in the high expression of CDCA8 and shorter overall survival (*P < *.0001, HR = 0.4270, 95% CI: 0.2630–0.6930, Fig. 2B). These results suggested that high expression of CDCA8 was associated with poor prognosis of BC patients.

### 4.4 Cox univariable and multivariable analyses of OS and CSS among BC patients

Cox univariable analyses showed intravesical therapy, N stage, tumor grade were the influence factors of overall survival and CDCA8 expression, intravesical therapy, tumor grade, progression were the influence factors of cancer specific survival among bladder cancer patients. But the cox multivariable analyses showed that intravesical therapy, N stage, progression were the independent influence factors of overall survival and CDCA8 expression, tumor grade, progression were the independent influence factors of cancer specific survival among bladder cancer patients (Tables 3 and 4).

**Table 3:**
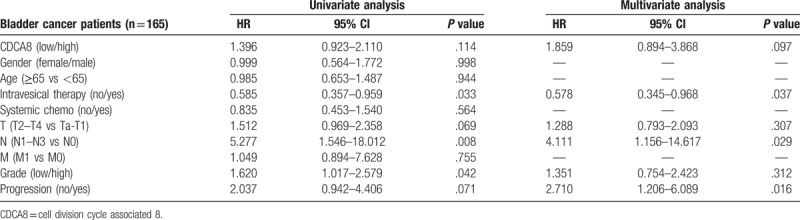
Cox univariable and multivariable analyses of overall survival among 165 bladder cancer patients.

**Table 4:**
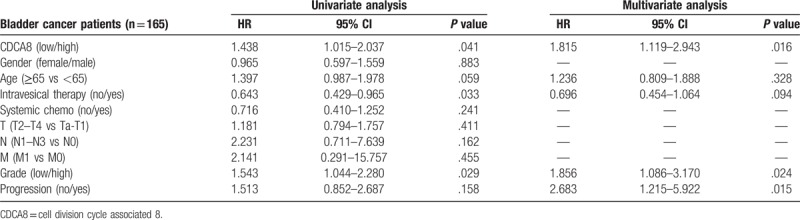
Cox univariable and multivariable analyses of cancer specific survival among 165 bladder cancer patients.

### 4.5 Pathways associated with CDCA8 expression

To investigate the CDCA8-related pathways that possibly affect the growth of BC cells, GSEA was carried out and “h.all.v6.0.symbols.gmt” was used as reference. The results of GSEA indicated that signaling pathways including “spermatogenesis,” “G2 M checkpoint”, “E2F targets,” “unfolded protein response,” “Myc targets V1,” “Myc targets V2,” “mTORC1 signaling,” “mitotic spindle,” “PI3K-AKT-mTOR signaling,” “cholesterol homeostasis,” and “glycolysis” were identified to be significantly altered along with CDCA8 aberrant expression (Table 5). GSEA demonstrated that CDCA8 played a role in the development of BC.

**Table 5:**
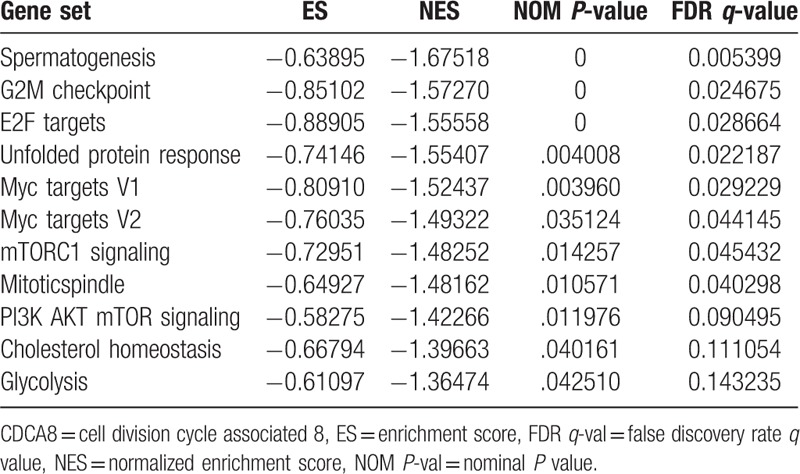
Pathways associated with CDCA8 expression.

### 4.6 Expression of CDCA8 by RT-PCR in BC tissues

**Figure 3:**
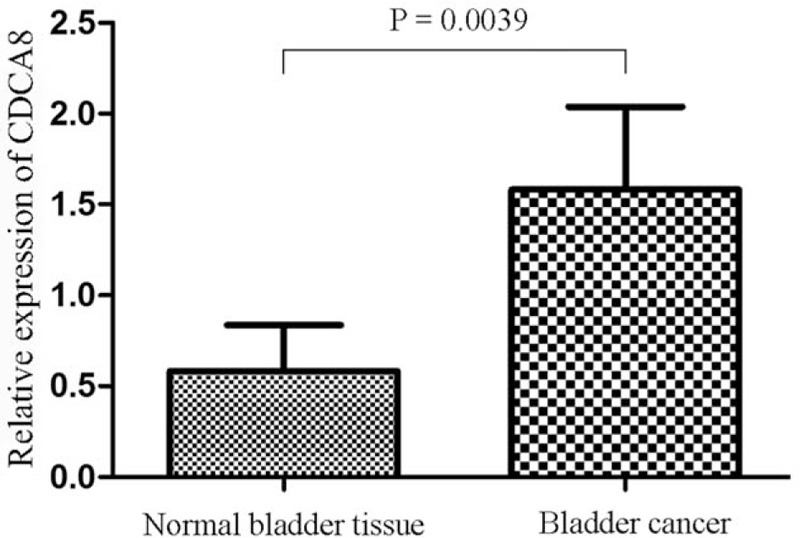
qRT-PCR analysis exhibited the expression of CDCA8 in bladder cancer tissues compared with the paired paracancerous tissues. CDCA8 = cell division cycle associated 8, qRT-PCR = quantitative real time-polymerase chain reaction.

RT-PCR results showed that CDCA8 expression was upregulated in 10 BC samples compared with the paired adjacent tissues. The mean expression levels of CDCA8 in cancer tissues were significantly higher than those in relevant normal tissues (*P = *.0039, Fig. 3).

## 5 Discussion

BC is one of the most common tumors in the urinary system and the prognosis of patients with BC remains poor. Histological tumor stage and grade are considered as 2 independent prognostic factors in the recurrence of BC.^[19]^ As previously reported, tumor stage and nodal involvement are independent prognostic variables of muscle invasive bladder cancer (MIBC) survival,^[20]^ and MIBC (Tstage≥T2) is generally associated with poor prognosis.^[21]^ The treatment and survival outcomes of nonmuscle invasive bladder cancer (NMIBC) and MIBC are obviously different. In this study, the chi-square test (Table 2) showed that T stage was associated with the expression of CDCA8. The higher the expression of CDCA8, the more the proportion of ≥T2 stage patients, which means the greater the risk of MIBC. This indicates that CDCA8 may be of some value in predicting the occurrence of MIBC, but more case analysis and long term follow-up are needed to verify it.

Dysregulation of the cell-cycle progression and uncontrolled cell proliferation are the hallmark of cancers.^[22]^ It is well acknowledged that CPC plays an important role in cell division. Walker^[23]^ identified CDCA8 as one of the components of CPC in mitosis. Loss of CDCA8 led to defective cell proliferation and early embryonic lethality.^[24]^ Previous studies revealed that CDCA8 expression was related with poor prognosis of patients with gastric cancer,^[25]^ lung cancer^[26]^ and hepatocellular carcinoma.^[27]^ Another study also showed that CDCA8 was overexpressed in colorectal cancers, and that loss of CDCA8 suppressed the growth of cancer cells and induced apoptosis.^[12]^

The high CDCA8 expression may be related to other CPC proteins. Previous studies suggested that CDCA8 interacted with Survivin to positively regulate the segregated chromatids and abnormal mitotic bridges.^[24]^ Moreover, disruption of the interaction between CDCA8 and Survivin inhibited the growth of hepatocellular carcinoma.^[28]^ A functional CPC required aurora B activity and INCENP, Survivin and CDCA8 for proper activation and localization of the kinase. In vitro, CDCA8, Survivin and INCENP were required for activation and/or localization of Aurora B. Our study and the previous studies suggested that Borealin could affect the growth and progression of cancer cells. However, the mechanisms remained to be investiagted.

## 6 Conclusions

In the present study, we found that CDCA8 was overexpressed in BC cells and affected the progression of BC in patients. Furthermore, overexpression of CDCA8 was associated with poor prognosis of BC patients. Taken together, our findings illustrated that CDCA8 was promising to act as a valuable biomarker for BC progression and a potential therapeutic target for BC treatment.

## Author contributions

Conceived and designed the experiments: SL. Performed the experiments: YB, SC, JJ, JY, GW and QZ. Analyzed and interpreted the data: YB and SC. Contributed reagents/materials/analysis tools: SL. Contributed to the writing of the manuscript: YB and SC. The authors declared that there were no conflicts of interest regarding the publication of this paper.

**Conceptualization:** Sheng Li.

**Data curation:** Qiong Ya Bi, Gang Wang.

**Formal analysis:** Qiong Ya Bi, Gang Wang.

**Funding acquisition:** Sheng Li.

**Investigation:** Song Chen, Jie Yao, Gang Wang.

**Methodology:** Song Chen, Jie Yao, Gang Wang, Qiang Zhou.

**Project administration:** Sheng Li.

**Resources:** Zhi Jia Jiang.

**Software:** Zhi Jia Jiang, Jie Yao.

**Supervision:** Song Chen, Zhi Jia Jiang, Jie Yao.

**Validation:** Song Chen, Qiang Zhou.

**Visualization:** Qiong Ya Bi, Song Chen, Qiang Zhou.

**Writing – original draft:** Qiong Ya Bi, Song Chen.

**Writing – review & editing:** Sheng Li.
